# From Orai to E-Cadherin: Subversion of Calcium Trafficking in Cancer to Drive Proliferation, Anoikis-Resistance, and Metastasis

**DOI:** 10.3390/biomedicines8060169

**Published:** 2020-06-21

**Authors:** Aarushi Sharma, Randolph C. Elble

**Affiliations:** 1 Department of Pharmacology, Southern Illinois University School of Medicine, Springfield, IL 62702, USA; sharma.aarushi1821@gmail.com; 2 Department of Pharmacology and Simmons Cancer Institute, Southern Illinois University School of Medicine, Springfield, IL 62702, USA

**Keywords:** calcium, SOCE, SICE, IP3R, Orai, Stim, EGFR, E-cadherin, IQGAP, calmodulin, anoikis, circulating tumor cells

## Abstract

The common currency of epithelial differentiation and homeostasis is calcium, stored primarily in the endoplasmic reticulum, rationed according to need, and replenished from the extracellular milieu via store-operated calcium entry (SOCE). This currency is disbursed by the IP3 receptor in response to diverse extracellular signals. The rate of release is governed by regulators of proliferation, autophagy, survival, and programmed cell death, the strength of the signal leading to different outcomes. Intracellular calcium acts chiefly through intermediates such as calmodulin that regulates growth factor receptors such as epidermal growth factor receptor (EGFR), actin polymerization, and adherens junction assembly and maintenance. Here we review this machinery and its role in differentiation, then consider how cancer cells subvert it to license proliferation, resist anoikis, and enable metastasis, either by modulating the level of intracellular calcium or its downstream targets or effectors such as EGFR, E-cadherin, IQGAP1, TMEM16A, CLCA2, and TRPA1. Implications are considered for the roles of E-cadherin and growth factor receptors in circulating tumor cells and metastasis. The discovery of novel, cell type-specific modulators and effectors of calcium signaling offers new possibilities for cancer chemotherapy.

## 1. Introduction

Calcium plays a unique role among all the ions that exist in the fluids in and around a living cell to maintain cellular homeostasis and other physiological functions [1]. Unlike sodium and potassium, calcium has a huge concentration gradient across cell membranes resulting from very low intracellular level of this cation [2]. All living cells maintain cytosolic calcium ion concentration in the range of 100 nM which is nearly 20,000-fold less than its extracellular level [3]. Early studies suggest this to be a mechanism adopted by cells to avoid stress by preventing precipitate formation with abundant phosphate anions that are required for energy production [3]. Consequently, most of the time low cytosolic calcium levels persist in cells which is leveraged in the form of small calcium ion fluxes creating greater cellular responses [2].

**Figure 1:**
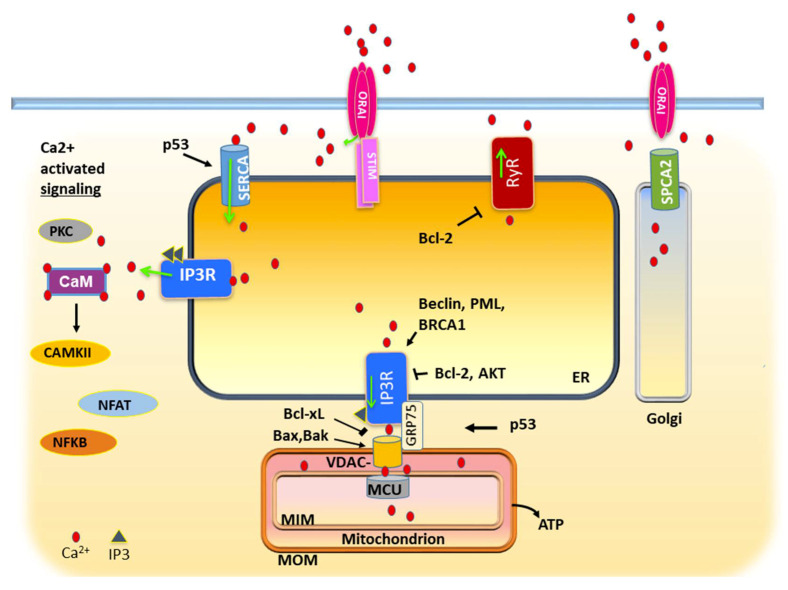
Storage, utilization, and renewal of intracellular calcium: a simplified view of core Ca^2+^ signaling machinery. Cytosolic Ca^2+^ is continuously sequestered into the ER by SERCA pumps. IP3 signals from certain cell-surface receptors (not shown) opens IP3R channels that direct Ca^2+^ to the cytosol or mitochondrion. Ca^2+^ -binding proteins in the cytosol then stimulate a host of cell type-dependent signaling pathways. In some cell types, the IP3R may co-exist and functionally overlap with another channel, the ryanodine receptor, RyR. Ca^2+^ transfer to mitochondrion is mediated by IP3R coupled to the mitochondrial channel VDAC by the adapter molecule GRP75. MCU then admits Ca^2+^ to the inner matrix, where it drives metabolism and ATP production. Calcium overload causes apoptosis by disrupting membrane potential and releasing cytochrome C; thus, Ca^2+^ transfer to mitochondria is tightly modulated, either positively or negatively, by regulators of proliferation, autophagy, and death such as the Bcl-2 family, Beclin, AKT, PML, p53, and BRCA1. Depletion of ER Ca^2+^ causes the Ca^2+^ sensor STIM1 to engage the plasma membrane Ca^2+^ channel Orai1, causing an influx of extracellular Ca^2+^ to the cytosol. SERCA pumps may associate with this complex, allowing more direct replenishment of ER Ca^2+^. The Golgi Ca^2+^ pump SPCA2 can also bind and activate Orai1 to drive epithelial differentiation. See text for details. ER, endoplasmic reticulum; MOM, mitochondrial outer membrane; MIM, mitochondrial inner membrane; MCU, mitochondrial calcium uniporter; VDAC, voltage-dependent anion channel. Black arrows indicate positive regulation and T-bar indicates negative regulation. Green arrows denote direction of flow.

As a potent signaling agent, Ca^2+^ must also be sequestered from its targets in the cytosol before the time of action. This is accomplished by ATP-driven SERCA (Sarco/ER Ca^2+^- ATPase) pumps in the membrane of the ER, the principal repository of iCa^2+^ (intracellular calcium; Figure 1) [4]. Release of Ca^2+^ from the ER is mediated by IP3 receptors, ligand-dependent Ca^2+^ channels that are regulated by a host of proteins in response to physiological cues [5,6]. Ca^2+^ may be released to the cytosol, to organelles such as the mitochondria, or to plasma membrane microdomains. Depletion of ER calcium reserves is detected by a membrane sensor that responds by triggering the opening of a plasma membrane Ca^2+^ channel (Orai1) in a process termed store-operated calcium entry (SOCE) [7]. In the first half of this review, we will describe how this machinery functions in normal epithelial cells and how that changes in carcinomas, focusing on storage, utilization, and replenishment. In the latter half, we consider its role in epithelial proliferation, differentiation, and stress response, and how that is exploited to promote metastasis.

## 2. Storage and Management of Intracellular Calcium in Normal and Cancer Cells

### 2.1. Intracellular Stores: IP3R, RyR, SERCA

Release of Ca^2+^ from the ER in epithelial cells is mediated primarily by a family of tetrameric channels termed inositol triphosphate receptors (IP3R 1−3) [5,6]. The IP3R opens in response to its ligand IP3, generated by phospholipase C (PLC) in response to diverse extrinsic stimuli [5]. IP3Rs are also co-activated by Ca^2+^, creating a positive feedback loop when IP3 signaling is strong [5]. This stream of Ca^2+^ ions may be directed to the cytosol or to the mitochondria or other intracellular microdomains (Figure 1).

The IP3R is a tightly regulated rheostat and a signaling nexus. Ca^2+^ released from the ER is required for normal metabolism and proliferation, and its level determines whether cells divide or undergo autophagy or apoptosis [8,9,10,11]. Not surprisingly, many regulators of these processes bind and modulate the IP3R. AKT, a kinase that transduces mitogenic and survival signals, ensures a homeostatic level of activity by binding and phosphorylating IP3Rs at a conserved site, serine 2681 [12]. This modification dampens proapoptotic Ca^2+^ transfer to mitochondria and ensures cell survival during stress. Unrestrained transfer results in Ca^2+^ overload, loss of mitochondrial outer membrane potential, mitochondrial outer membrane permeabilization (MOMP), and escape of cytochrome C to initiate the intrinsic apoptotic cascade [13]. AKT activity is frequently upregulated in cancer, either by activating mutations in its kinase domain or loss of negative regulation upstream by deletion of PTEN [14].

Other suppressors of apoptosis, Bcl2, Bcl-xL, and Mcl1, also bind to IP3Rs [15,16,17,18,19,20]. Although better known for their role in blocking the action of apoptogenic BH3-containing proteins in the intrinsic apoptotic cascade, the Bcl2 family plays an equally important role in modulating Ca^2+^ efflux from the ER [21]. Bcl2 itself dampens transfer by binding to the VDAC channel on the mitochondrial surface that receives the Ca^2+^ flux [22,23]. At the IP3R, its action is less clear. It has been reported to either inhibit high level Ca^2+^ flux or promote continuous leakage that reduces ER stores to nonthreatening levels [21]. The latter function has also been attributed to a distinct apoptosis inhibitor, Bax inhibitor-1 [24]. Overexpression of all three Bcl-2 family members sensitizes the IP3R to IP3 and enhance Ca^2+^ oscillations, but each has its own specializations. For example, Bcl-xL seems more important for maintaining normal metabolism, and its interaction with BH3-like domains in IP3R is required for viability, while Mcl-1 is required for maintaining ER structure [19,25,26]. All three are often upregulated in cancers, especially leukemias, and inhibitors such as venetoclax have greatly improved survival in CLL [21,27]. In fact, venetoclax is now approved as first-line therapy. In clinical trials, many patients experienced complete response and remained in remission following cessation of the drug. Incredibly, in combination with the Bruton’s kinase inhibitor ibrutinib, nearly all patients had complete response with no detectable residual disease.

In addition to these tumor-promoting proteins, tumor suppressors also regulate IP3R activity. For example, among the many activities of the PML protein, it recruits PP2A (protein phosphatase 2A) to IP3Rs where it antagonizes the effects of AKT [28]. Loss of PML in cancer prevents the high level of Ca^2+^ transfer to mitochondria required for apoptosis [28]. At late stages of apoptosis, cytochrome C released from mitochondria binds to the C-terminal segment of the IP3R and forces it open to ensure Ca^2+^ overload [29]. The BRCA1 protein binds to the IP3R in response to DNA damage and sensitizes it to IP3, resulting in heightened levels of mitochondrial Ca^2+^ [30]. Even under non-stressed conditions, a pool of BRCA1 is associated with the ER membrane through a lipophilic domain.

Inducers of autophagy also impinge on the IP3R. The interactor Beclin1 is required for nucleating the autophagosome and is negatively regulated by AKT and Bcl2 [31]. Thus, the IP3R was proposed to function merely as a signaling scaffold for negative regulation of autophagy. However, in other studies, induction of autophagy by starvation was associated with increased Beclin1 binding to IP3R and an increase in Ca^2+^ flux that seemed to be required for completion of autophagy [21,32]. In considering these conflicting data, it should be kept in mind that there are three IP3R isoforms, and they may differ in interactions and localization or function. Bultynck has suggested that IP3R3 may be more involved in mitochondrial interaction, while IP3R1 may chiefly mediate efflux to the cytosol [33].

A low-level, constitutive flow of Ca^2+^ through the IP3R to the mitochondrion is essential for normal metabolism [8,34]. The conduit for Ca^2+^ transmission from the ER to the mitochondrion is formed by linkage of the IP3R to the mitochondrial channel VDAC1 by the adaptor protein GRP75 ([35]; Figure 1). Transmitted Ca^2+^ stimulates dehydrogenases in the TCA cycle and promotes oxidative phosphorylation leading to ATP synthesis [36,37]. Cardenas and coworkers found that blocking this transmission with the pan-IP3R inhibitor Xestospongin B (XeB) caused ATP levels to drop, and AMPK-dependent autophagy ensued [38]. Intriguingly, autophagy permitted survival of normal but not cancer cells of prostate or breast, which underwent mitotic catastrophe and necrosis. Cancer cells could be partly rescued by supplementing media with nucleosides, suggesting a bioenergetic and metabolic deficit since nucleotide synthesis depends on the TCA cycle in functional mitochondria. Unlike normal cells, cancer cells were unable to delay entry into S phase, and incomplete DNA synthesis in the absence of cell cycle checkpoints resulted in mitotic catastrophe. Therapeutically encouraging, XeB was also effective in inhibiting growth of melanoma xenografts in mice.

A second class of Ca^2+^- release channel termed ryanodine receptors (RyR) exists in excitable cells, but they are also found in some epithelial tissues and are sometimes greatly upregulated in diverse cancers (Figure 1) [39]. In keratinocytes, they are associated with differentiation and cell–cell barrier formation [40]. Like the IP3Rs, RyR1-3 are Ca^2+^- activated, redox-sensitive channels with multiple protein partners that modulate their activity [38]. One partner they share with IP3Rs is Bcl-2 [41]. Bcl-2 binds to and inhibits Ca^2+^ release by ryanodine receptors. However, RyRs are frequently upregulated in breast cancer, and their expression level correlates with tumor grade [42,43,44]. Like IP3Rs, they also have a complex interrelationship with mitochondria [45]. Reactive oxygen species released by mitochondria oxidizes and activates the RyR. Despite those similarities, it should be said that RyRs and IP3Rs are structurally distinct proteins, having only 17% sequence identity [46]. RyRs are sensitive to different ligands such as cyclic ADP-ribose and can be activated by Ca^2+^ alone [47,48]. They also differ in their relation to autophagy. While both IP3Rs and RyRs can suppress basal autophagy, they act at a different level. IP3Rs suppress autophagy at a proximal level by driving mitochondrial bioenergetics and thus decreasing AMPK activity, while RyRs block autophagy at a distal level by counteracting the fusion of autophagosomes and lysosomes [49]. Nevertheless, the overlaps in function, regulation, and expression of RyRs and IP3Rs in the same cell type underscore the complexity of Ca^2+^ signaling in the cytosol and between intracellular compartments. Thus, it may be risky to draw conclusions about the role of Ca^2+^ in physiology of a tumor cell based on the expression or activity of one such channel alone.

Ca^2+^ sequestration in the ER by SERCA pumps is a regulated process that is frequently disrupted in cancer cells. In response to stress, p53 activates SERCA pumps to increase the Ca^2+^ content of the ER and enhances membrane interactions between the ER and mitochondrion (Figure 1) [50]. Until p53 is inactivated, this Ca^2+^ charge is a threat to the survival of the tumor cell. Indeed, SERCA overexpression was found to sensitize cells to chemotherapy-induced apoptosis [50]. Therefore, it was proposed that early in tumorigenesis, tumor cells should downregulate SERCA pumps to prevent overload [51]. Accordingly, downregulation of SERCA2 was observed in lung and colon cancers and cell lines [52,53,54]. SERCA3 was reported to be upregulated during differentiation in the colon and progressively downregulated in adenomas and adenocarcinomas and in leukemia cell lines [54,55,56]. There are exceptions however. In Notch-1-dependent leukemia, SERCA activity was found to be required for progress of the cell cycle [57]. The role of SERCA pumps in a particular cancer cell may depend upon its developmental history and the constellation of mutations that together determine whether ER Ca^2+^ stores are a threat or a survival tool. Early activation of, and dependence upon, a Ca^2+^- dependent growth factor signaling pathway, for example, could force adaptations such as Bcl-2 overexpression to inhibit mitochondrial Ca^2+^ surge and thus allow higher levels of expression of SERCA pumps. These adaptations could allow a nascent cancer cell to maintain and exploit some aspects of the differentiation program without provoking apoptosis. Cancer stage and SERCA isoform switching may also come into play, as observed with Golgi Ca^2+^ pumps in breast cancer [51,58]. The application of SERCA pump inhibitors to cancer therapy has been limited because of their toxicity to normal tissues. This problem has been circumvented by conjugating a thapsigargin analog to a peptide that binds a prostate-specific receptor [59]. The drug, G202, was effective in vitro and now is in clinical trials for prostate cancer. Other SERCA inhibitors have been found effective against a variety of solid tumor types in preclinical studies (see Section 7).

Not all calcium trafficking to and from the ER is actively regulated. Under resting conditions, influx via SERCA is balanced by passive leakage through ribosomal translocons [60]. This could be demonstrated using drugs that locked the translocon into a closed or open position. Physiologically, under normal conditions the stress-responsive chaperone GRP78 blocked translocons and prevented leakage, while stress triggered its release and increased leakage [61]. Rather than a design flaw, passive leakage appears to be an integral feature of Ca^2+^ homeostasis.

In addition to the ER-Golgi and mitochondrion, Ca^2+^ stored in the lysosome is also physiologically important. In addition to its roles in protein recycling and autophagy, the lysosome is indispensable for certain aspects of cell migration, and lysosomal Ca^2+^ stores are essential. Lysosomal Ca^2+^ is regulated by different families of channels than are present in the ER, and their expression is often dysregulated in cancer, reviewed by Sterea et al. [62]. Recently Ca^2+^ released from the lysosome by one such channel, Mucolipin 1 (MCOLN1), was found to play an important role in autophagy by activating the phosphatase calcineurin, which was essential for nuclear translocation of transcription factor TFEB, an activator of genes involved in autophagy and lysosome biogenesis [63]. MCOLN1-released Ca^2+^ is essential for interaction of lysosomes to the dynein-linked intracellular translocation network and for intermembrane fusion [64].

### 2.2. Store-Operated Calcium Entry

The role of SOCE in cancer has been described recently in some excellent reviews [51,65,66,67]. Here we highlight the major findings that establish the signaling environment in adenocarcinomas and are relevant to anoikis resistance, EGFR signaling, and CTCs.

In most normal cells, exhaustion of ER Ca^2+^ reserves is detected by the transmembrane Ca2+ sensor STIM1 which responds by binding and activating the plasma membrane channel Orai1, which floods the local cytosol with Ca^2+^. Most is then sequestered by SERCA pumps in the closely apposed ER membrane (Figure 1) [51]. Because of the role of Ca^2+^ in apoptosis, it has been proposed that cancer cells early in progression may find advantage in minimizing SOCE, while later in progression after inactivation of apoptotic signaling by p53, they may upregulate Ca^2+^ influx to benefit from its metabolic and mitogenic effects [51]. As most cancer cell lines are derived from aggressive tumors, STIM and Orai expression is usually increased [51]. For example, in breast cancer, glioblastoma, renal cell carcinoma, and esophageal squamous cell carcinoma (ESCC), STIM1 and Orai1 are reported to contribute to proliferation, migration, or both [68,69,70,71]. In ESCC, elevated expression of Orai1 in tumors was associated with poor prognosis, and its inhibition in cell lines by siRNA or drugs reduced SOCE and suppressed proliferation, invasion, and growth of tumor xenografts [72]. ESCC cells with high Orai1 exhibited an increase in cytosolic Ca^2+^ oscillations, consistent with activation of a few localized IP3Rs, suggested by the authors in this case to result from EGF binding to EGFR [72].

On the other hand, in prostate cancer, Orai1-mediated SOCE sensitized cells to apoptosis in anti-androgen-resistant cells [73]. However, prostate cancer cells also often overexpress Orai3, an alternative Orai isoform that can be store-dependent or independent. Orai3 can be activated by arachidonic acid, production of which is upregulated during tumor progression [74]. Orai3 forms heteromers with Orai1, reducing SOCE and levels of ER Ca^2+^ and protecting from apoptogenic levels of Ca^2+^ transfer to mitochondria, while increasing cytosolic Ca_2+_ levels [75].

The Orai1 inhibitor RP4010 showed therapeutic promise in preclinical work, but a clinical trial in patients with refractory or relapsed lymphoma (NCT03119467) has been terminated because of safety concerns [76]. Some other, already FDA-approved drugs have been found to inhibit SOCE and may be repurposed [77].

### 2.3. Store-Independent Calcium Entry

**Figure 2:**
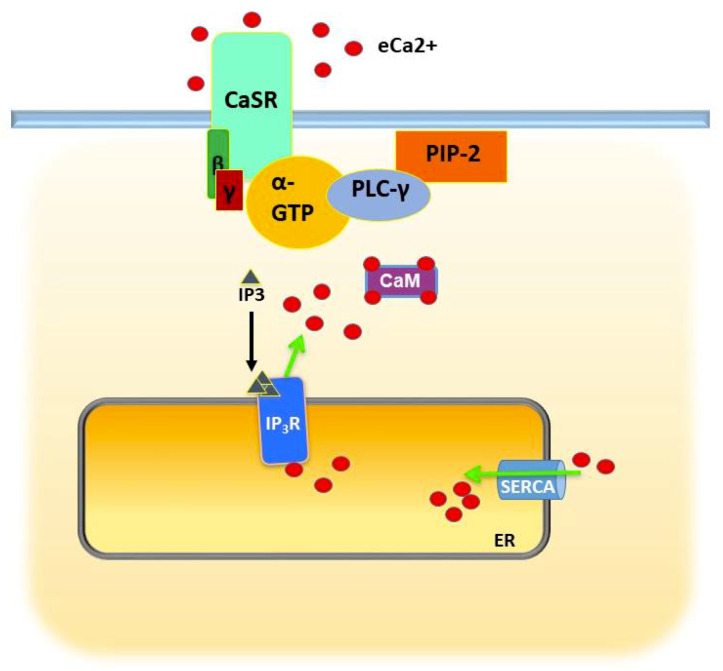
Role of calcium-sensing receptor (CaSR) in detecting extracellular I Ca^2+^ and signaling the release of intracellular (i) Ca^2+^ in response. Binding of eCa^2+^ to CaSR stimulates PLC gamma to generate IP3 which then binds to its receptor channel on the ER, triggering release of sequestered Ca^2+^. Cytosolic Ca^2+^ can then activate various signaling pathways by binding to CaM or other calcium-binding proteins. Excess Ca^2+^ is resequestered by SERCA pumps. Green arrow indicates direction of flow. Black arrow indicates activation.

Another route to store-independent activation of Orai1 is through the Golgi which, like the ER, sequesters Ca^2+^. There, the role of SERCA pumps is played by secretory pathway Ca^2+^- ATPase isoforms SPCA1 and SPCA2 [78]. SPCA1 is ubiquitous and essential, while SPCA2 is limited to tetrapod epithelia. Both are overexpressed in certain cancers but with a different distribution. Rao and coworkers showed that in breast cancer, SPCA1 is associated with cell lines having a basal or mesenchymal phenotype, typified by N-cadherin expression, while SPCA2 is associated with an epithelial profile and correlates with E-cadherin expression [79,80,81]. SPCA2 can activate store-independent Ca^2+^ entry (SICE) into the cytosol by binding directly to Orai1 [81]. This ability to activate Orai1 does not depend on the Ca^2+^ pumping activity of SPCA2 or on Golgi Ca^2+^ stores [82]. Knockdown (KD) of SPCA2 inhibits surface expression of E-cadherin, cell–cell adhesion, and the Hippo-YAP pathway [79]. SPCA1 lacks these properties. Rao has proposed that in normal epithelial cells, SICE mediated by SPCA2-Orai1 is balanced by Ca^2+^ egress via ATP-driven Ca^2+^ pumps in the plasma membrane (PMCA); the resultant level of cytosolic Ca^2+^ is optimized for epithelial differentiation processes [78]. However, in luminal breast cancer cells PMCA pumps are downregulated, and heightened Ca^2+^ concentration instead drives cell proliferation [80]. The differing effects of SPCA expression in different breast cancer subtypes suggests that any therapeutic strategies would have to be carefully targeted. (For a comprehensive listing of mutational effects on calcium signaling apparatus in various cancers, see ref. [51], Tables 2 and 3; ref. [80], Table 2; and ref. [66], Figure 2.)

## 3. Calcium Regulation of Epithelial Differentiation, E-Cadherin-Beta Catenin, and Cell–Cell Adhesion

The requirement for eCa^2+^ in epithelial differentiation was appreciated early in the development of cell culture of keratinocytes [83]. While cells proliferated at concentrations less than 50–70 micromolar, higher concentrations arrested proliferation and promoted terminal differentiation. Certain cell–cell adhesion and signaling proteins, later christened cadherins, turned out to depend on both e(extracellular)Ca^2+^ and iCa^2+^ for their function [84,85,86]. Increases in eCa^2+^ triggered increases in iCa^2+^ via activation of the calcium-sensing receptor (CaSR), a G-protein-coupled receptor [87,88,89] that signals via phospholipase C (PLC) gamma and IP3 to stimulate release of Ca^2+^ via the IP3R from the ER [90] (Figure 2). iCa^2+^ was found to be required for differentiation, as chelation of iCa^2+^ using BAPTA-AM prevented aspects of differentiation such as adherens junction (AJ) formation, resulting in diffused cytoplasmic or membrane staining of E-cadherin, impaired tight junction formation, and reduced epithelial keratin expression [86]. To distinguish between the effects of cytosolic Ca^2+^ and stored calcium, Li et al. varied eCa^2+^ in the presence of the SERCA pump inhibitors thapsigargin and CPA [91]. They found that assembly of cell–cell junctions depended on sustained increases of iCa^2+^; transient release from intracellular stores was insufficient without replenishment from the extracellular space.

**Figure 3:**
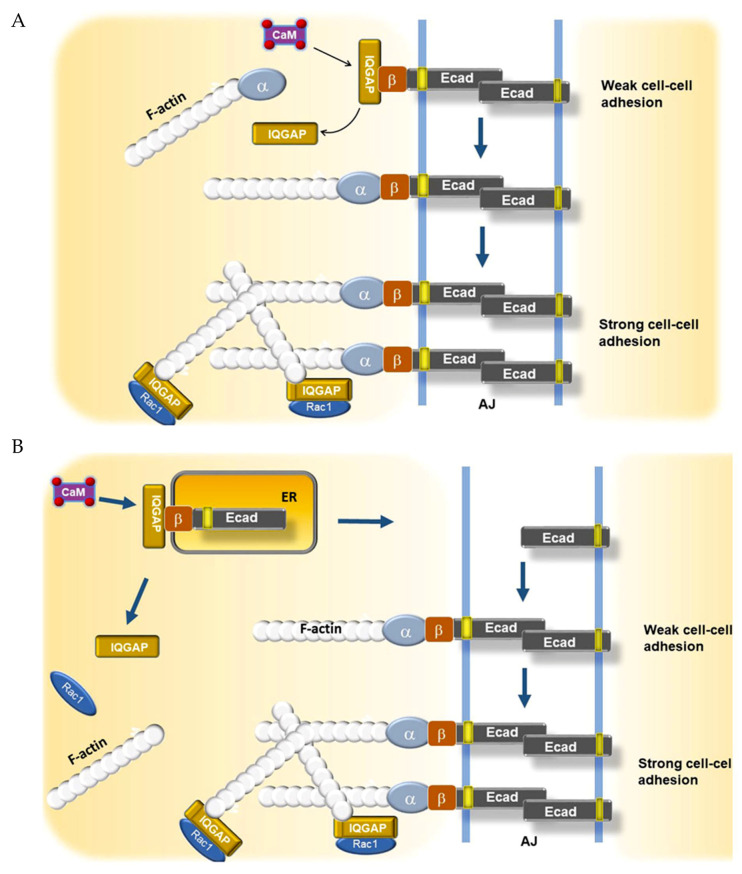
Two models for the role of Ca^2+^/CaM and IQGAP1 in adherens junction (AJ) formation and maturation. (**A**), IQGAP-bound Ecad-beta catenin can translocate to the cell–cell junction but form only a weak adhesion without displacement of IQGAP. Ca^2+^/CaM displaces IQGAP1 from beta catenin-E-cadherin, allowing association of an alpha catenin-bound actin microfilament with the nascent AJ. In the presence of activated GTP-bound Rac1 or CDC42, IQGAP promotes formation of an actin meshwork, leading to strong anchoring and cell–cell adhesion. (**B**), the IQGAP1-beta catenin-E-cadherin complex is retained in the ER until Ca^2+^/CaM displaces IQGAP1 at the ER membrane, allowing vesicle transport from the ER-Golgi to the junctional plasma membrane, immediately followed by the association of an alpha catenin-bound actin microfilament with the nascent AJ. Beta, beta catenin; alpha, alpha catenin; Ecad, E-cadherin. Red dots, calcium. For simplicity, Ecad is represented as a monomer. Based on work of Li, Noritake, Suisse, and co-workers [86,91,94,96].

How then does iCa^2+^ influence E-cadherin surface localization? As E-cadherin is translated into the ER, it is assembled into a complex with beta catenin, and this complex is bound by IQGAP1 (IQ motif-containing GTPase-activating protein 1), a scaffold protein with a plethora of interactors [92,93,94]. As described earlier, iCa^2+^ signals are usually transduced via the calcium-binding protein CaM. Noritake et al. reported that Ca^2+^/CaM competes with E-cadherin-beta catenin for binding to IQGAP1 [94]. They found in MDCK cells that IQGAP1-bound E-cadherin-beta catenin-containing vesicles could translocate to cell–cell junctions but mediate only weak adhesion in the absence of Ca^2+^/CaM because the complex was unable to recruit alpha catenin and engage the actin cytoskeleton. Ca^2+^/CaM binding displaced IQGAP1 from this complex, allowing alpha catenin to connect beta catenin to actin filaments (Figure 3A). IQGAP1 also helps to anchor the AJ to the cytoskeleton through its interactions with actin and CDC42/Rac-GTP, which induce formation of an actin meshwork, resulting in strong cell–cell adhesion [95].

Much more recently, other workers have reported that translocation of E-cadherin-beta catenin to the plasma membrane is vitally dependent on ER Ca^2+^. Suisse and Treisman investigated this question utilizing hypomorphic and null SERCA and Orai mutants in Drosophila [96]. They used the mutants to establish high, intermediate, or low levels of ER Ca^2+^ and measured their effects on E-cadherin localization. In high Ca^2+^, most E-cadherin-beta catenin appeared at the cell–cell junction with little in the ER. Moderately impairing SERCA function either mutationally or chemically reversed this situation, with the lion’s share of the complex retained in the ER (Figure 3B). Fuller reduction of the ER Ca^2+^ trapped all of the complex in the ER. Thus, details of this pathway may vary with species, cell type, and the level of iCa^2+^.

Calcium also drives other events at cell–cell junctions. Stuart et al. reported that loss of iCa^2+^ inhibited sorting of junctional proteins ZO-1 and desmoplakin to cell–cell junctions and blocked formation of tight junctions and desmosomes in MDCK cells [97]. Jouret et al. observed that TJ formation and barrier activity were dependent on CaSR-stimulated iCa^2+^ and could be blocked by BAPTA-AM [98]. CaSR agonists stimulated occludin migration to cell–cell junctions and association of ZO-1 TJ adaptor protein with actin binding protein Afadin. This study also revealed that CaSR expression is stimulated by calcium and that the protein localizes to basolateral junctions, further cementing the critical role of both eCa^2+^ and iCa^2+^ in epithelial tissue generation and homeostasis.

## 4. Calcium Regulation of EGFR

EGFR, also known as Her1, is a member of a family of four plasma membrane receptor-tyrosine kinases termed Her1-4 that play essential roles in development, proliferation, and differentiation in a multiplicity of tissues [99,100,101,102,103,104]. EGFR binds several structurally similar growth factor ligands that trigger conformational changes in the ectodomain to activate a tyrosine kinase domain on the cytoplasmic face, resulting in autophosphorylation of several tyrosines [101,102,103]. This allows binding of a panoply of proteins that initiate multiple signaling cascades activating PI3K, MAPK, and STAT3 [102]. EGFR is often upregulated or constitutively activated in carcinomas and glioblastomas, and this presages a poor outcome in some organ sites [105,106]. EGFR-specific tyrosine kinase inhibitors (EGFRi) have been developed that improve overall survival for patients whose tumors are addicted to EGFR signaling for proliferation and survival [107]. However, for certain cancers it is very difficult a priori to determine which cancers will respond [107,108]. The need for biomarkers of EGFR-dependence has ignited a renewed interest in EGFR signaling mechanisms. Such markers might not only allow precision targeting of EGFRi but also reveal new therapeutic targets to enhance efficacy of EGFRi.

### 4.1. Calcium Regulation of EGFR via Calmodulin

**Figure 4:**
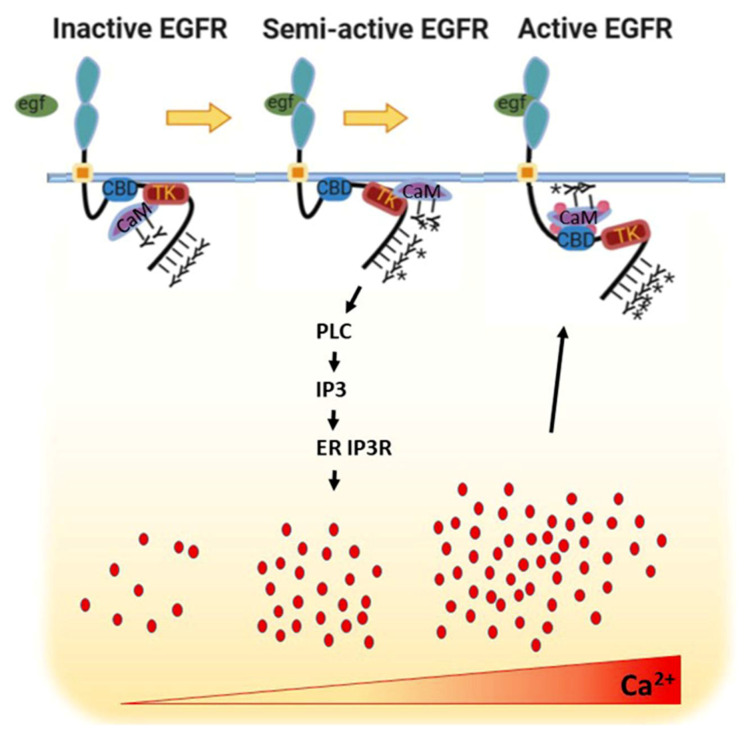
Model for sequential activation of EGFR by EGF, iCa^2+^, and CaM. Ligand binding results in partial activation of tyrosine kinase (TK) domain, phosphorylation of apo-CaM, and generation of IP3 by PLC gamma. Following release of Ca^2+^ from the ER, CaM binds Ca^2+^ and interacts tightly with the CaM-binding domain (CBD) of EGFR, dislodging it from its electrostatic interaction with the plasma membrane and permitting full activation of the TK domain. EGFR is depicted as a monomer for simplicity. Y *, phosphotyrosine. Red dots represent Ca^2+^. Adapted from Stateva et al. [110].

Calcium plays both positive and negative roles in modulating EGFR. The role of iCa2+ in activation of EGFR has been explored perhaps in most depth by Villalobo and coworkers [109,110]. They found that both iCa^2+^ and phospho-calmodulin (CaM) were required for full activation of EGFR by EGF [109,110]. Their data and that of McLaughlin et al. [111] showed that binding of calcium-phospho-CaM to a positively charged segment of the EGFR cytoplasmic domain separated the segment from the inner leaf of the plasma membrane, resulting in a conformational shift that allowed full autophosphorylation of EGFR. Mutation of the segment by alanine substitution prevented activation. Synthesizing a large body of biochemical data, they proposed the following model (Figure 4) [110]. Ligation of EGFR partially activates tyrosine-kinase activity resulting in phosphorylation of bound apo-CaM, partial phosphorylation of EGFR, and activation of PLC gamma. Production of IP3 and subsequent release of ER calcium stores via the IP3R results in calcium charging of CaM, which then binds the positively charged segment and allows full activation of EGFR. Thus iCa^2+^ modulates a positive feedback loop. Similar results were obtained for Her2 [112]. Thus, the ErbB family is calcium-dependent.

### 4.2. Calcium Regulation of EGFR via TMEM16A

**Figure 5:**
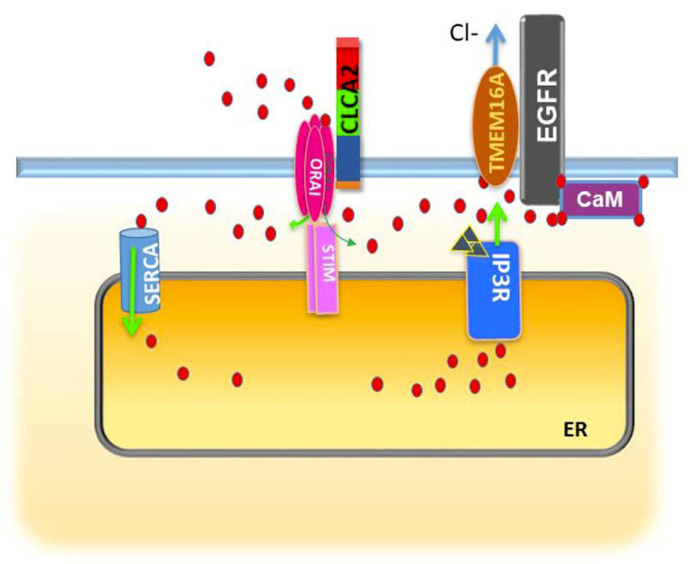
Model showing how CLCA2 might cooperate with TMEM16A to promote EGFR activation. CLCA2 enhances both SOCE via Orai1 and subsequently intracellular stores via SERCA, leading to greater IP3R-mediated Ca^2+^ release and activation of both TMEM16A and EGFR. At the same time, TMEM16A enhances EGFR activity by stabilizing it at the cell surface. EGFR, TMEM16A, and CLCA2 are depicted as monomers for simplicity. Arrows indicate direction of flow.

Another calcium effector, the calcium-activated chloride channel TMEM16A plays a major role in activating EGFR, and this property is exploited by several tumor types. In squamous cancers of head and neck, HNSCC, TMEM16A is frequently upregulated and correlates with metastasis [113,114,115], while KD in cell lines inhibits migration [115]. TMEM16A is also upregulated in a subset of other tumors and cell lines, especially those that upregulate EGFR and Her2 [116]. Knockdown in cell lines inhibited proliferation by downregulating EGFR and its downstream effectors AKT, ERK, CaMKII, and Src [116]. It consequently sensitized tumor cells to a panel of EGFR tyrosine-kinase inhibitors. This dependency of EGFR on TMEM16A was also observed in HNSCC, wherein inhibitors of TMEM16A synergized with EGFR inhibitors to limit tumor growth [117]. EGFR and TMEM16A were found to stabilize each other by forming a complex via their transmembrane regions (Figure 5). The level of TMEM16A upregulation correlated with sensitivity to EGFR inhibitors, with an r value of −0.8, while EGFR overexpression alone was not predictive. Thus, TMEM16A upregulation marks tumor cells, and possibly patient tumors, that are mostly likely to respond to these inhibitors.

An unexpected nuance to this exciting story was revealed in a subsequent study by some members of the same group [118]. They found that the most effective inhibitor of TMEM16A-mediated proliferation affected not only TMEM16A channel activity per se but also stability of the protein at the plasma membrane. Their results suggested that the anti-proliferative effect of the drug was due to loss of the protein, not just to loss of its chloride conductance. The authors concluded that TMEM16A has additional tumor-promoting activities beyond its conductance function.

TMEM16A function in turn is dependent on calcium and the proteins that regulate its intracellular availability. Multiple studies have shown that TMEM16A is activated by calcium either released through the closely apposed IP3R or via SOCE [119,120]. The CLCA family of calcium-activated chloride accessory proteins was recently shown to promote activation of TMEM16A by these mechanisms. Overexpression of CLCA2 enhanced chloride conductance by TMEM16A and enhanced both intracellular stores and SOCE [121]. CLCA2 was found to bind the Orai1/Stim1 complex that mediates SOCE in response to depletion of ER calcium stores. Subsequent refilling of the stores would then allow IP3R-mediated activation of TMEM16A (Figure 5). Tumor cell lines that upregulate CLCA2 also upregulate TMEM16A and EGFR, suggesting TMEM16A and CLCA2 are part of an EGFR-promoting axis.

The mechanisms by which EGFR is regulated by calcium and its modulators may vary between tissue and cancer types. Crottes et al. examined the relationship between TMEM16A, iCa^2+^ and EGFR in pancreatic ductal adenocarcinoma, PDAC [122]. They found that TMEM16A overexpression correlated with EGFR pathway upregulation and poor prognosis as in other cancers, and, as expected, treating cells with EGF induced the release of intracellular calcium, TMEM16A-mediated chloride current, and SOCE mediated by Orai1 and TRPC1. In addition, the cells became more persistently motile. Surprisingly however, TMEM16A KD or treatment with three different channel inhibitors blocked all of these effects. Thus, it seems that calcium mobilization from both within and without depends on chloride channel activity. A caveat here is that the inhibitors were not very specific. Niclosamide for example is sold as a STAT3 inhibitor and an antiparasitic [123]. In contrast to breast and HNSCC, KD of TMEM16A in the PDAC cells did not affect EGFR surface localization or endocytosis, but did reduce its phosphorylation at two tyrosines associated with activation. Notably, in these cells neither EGF nor TMEM16A KD had any effect on activation of ERK, suggesting ERK was already maximally activated by an unknown linked pathway. AKT was activated by EGF but this was little affected by KD. Instead, KD produced manifold changes in both the EGF-dependent and EGF-independent phosphoproteomes, especially of proteins involved in cell motility. These results suggest that TMEM16A expression primes PDAC tumor cells to exploit EGF signaling for metastatic spread. TMEM16A activation of EGFR seemed to be independent of calcium. The authors determined by proximity ligation assay that TMEM16A and EGFR did form a complex as observed in other cell types. Left unsettled was the question how TMEM16A activation might lead to both release of intracellular calcium and SOCE. Because only a single PDAC cell line was employed, it is unclear whether these findings will be generally true of PDAC. This study reinforces that TMEM16A, EGFR, and calcium are part of a regulatory axis that may be more effectively quashed by targeting multiple points in combination. How that can best be done will depend on the signaling pathways available in each tissue and how they are exploited in tumor initiation and progression.

## 5. Calcium, E-Cadherin, EGFR, and Routes to Anoikis-Resistance in Cancer

### 5.1. EMT vs. Retention of Epithelial Program in Circulating Tumor Cells

To depart the confines of the primary tissue, current models posit that aspiring cancer cells must reverse calcium-induced terminal differentiation, disassemble junctional anchorages, become resistant to detachment-induced apoptosis (anoikis), and activate a migratory program. To do this, they activate an embryonic and wound-healing program known as epithelial to mesenchymal transition that at once confers invasiveness, anoikis-resistance, and stem-like properties such as tumor-initiating potential [124,125,126]. It has been thought that metastatic cells reversed this process at distant organ sites to form new tumors [127]. While a vast body of science supports this view, the model is undergoing revision based on newer investigations of circulating tumor cells (CTCs) and anoikis-resistance mechanisms. Although most CTCs are single cells with a mesenchymal profile, evidence has emerged that the small percentage of clustered cells that retain the epithelial program are much more successful at forming new tumors at distant sites [128,129,130,131]. Transcriptional profiles have revealed that such clusters are enriched in epithelial junctional markers such as the AJ protein plakoglobin [128]. The presence of such clusters correlates with metastasis and poor prognosis [131].

### 5.2. E-Cadherin Cooperativity with EGFR and Her2 in Anoikis-Resistance

E-cadherin-mediated cell clustering is an alternate route to anoikis-resistance, and the mechanisms are gradually coming to light. In detached oral or skin squamous cells E-cadherin ligation stabilizes and activates EGFR and MAPK signaling in a ligand-independent manner [132,133]. Similarly, Schafer and co-workers found that in Her2+ mammary epithelial cells, aggregation of detached cells mediated by E-cadherin protected cells from anoikis by activating EGFR and survival signaling [134]. Interestingly, they found that detached cells were much more sensitive to the EGFR inhibitor gefitinib, implying that detached cells are more narrowly dependent on EGFR than attached cells, which receive survival signals from multiple pathways. This implies that EGFR inhibitors may be more effective at suppressing dispersal than primary tumor growth.

### 5.3. Detachment, iCa^2+^, and ROS in Anoikis-Resistance

As described earlier, these events all depend upon iCa^2+^. Earlier studies by Li et al. found that detachment of normal keratinocytes induced a steep rise in cytosolic calcium from both intracellular and extracellular sources, and that rise was required for subsequent induction of epithelial differentiation markers [91,135]. But what is driving this rise in iCa^2+^? The answer to this question has emerged from studies of altered metabolism of detached cells. In normal cells, loss of matrix-derived, integrin-mediated PI3K signaling reduces glucose import, leading to lower levels of NADPH and consequently higher ROS [136,137]. There is a complex interplay between regulators of ROS and iCa^2+^, comprehensively reviewed by Hempel and Trebak [138]. ROS increases iCa^2+^ through a variety of redox-sensitive mechanisms, and cancer cells often upregulate such mechanisms to avoid ROS-induced programmed cell death [138]. An example is the TRPA1 calcium channel. Cysteine-oxidation by ROS opens the channel, stimulating Ca^2+^/CaM-dependent activation of PYK2 and subsequent activation of Ras, PI3K, and downstream survival signaling [139]. Thus, TRPA1 may be a target for antimetastatic therapies based on calcium signaling inhibition. Another member of the TRP family, TRPV6, is also frequently upregulated in tumors of epithelial origin and correlates with progression [140]. An inhibitor is in clinical trials for multiple cancers and appears to be well tolerated [141].

Alternatively, cancer cells can survive detachment by overexpressing or mutationally activating growth factor receptors such as EGFR and Her2 to restore PI3K activity, glucose transport, and low ROS, among manifold other effects [136]. Viability of detached normal cells can be maintained experimentally by treating cells with antioxidants such as NAC even without restoration of glucose transport [135,136,137]. In cancer cells, the effect of antioxidants varies with the type of antioxidant and the genetic context. For example, the survival of detached K-Ras mutant tumor cells depends upon mitochondrially generated ROS and is inhibited by antioxidants [142]. However, in many cancers, including melanoma and lung, antioxidants such as NAC and vitamin E appear to enhance survival of metastatic cells and colonization of distant organs [143,144,145]. In an aging mouse model, NAC alone induced lung adenocarcinoma in 10% of normal mice and 50% of *JunD* mutant mice [146]. Similarly, breast cancers upregulate antioxidant mechanisms to evade anoikis, and inhibition of such mechanisms has been suggested as an approach to blocking metastasis [147]. However, neither antioxidants nor calcium supplements have fared well in clinical trials. In the SELECT trial, vitamin E supplements actually increased prostate cancer by 17% while having no effect on incidence of other major cancers [148]. Similarly, calcium/vitamin D supplements had no significant effect on incidence of any major cancer except for increasing incidence of precancerous polyps by fourfold, opposite to the expectation of the study designers [149,150]. These observations underscore the hazard of untargeted dietary antioxidant or calcium supplementation in an aging population.

## 6. E-Cadherin, Invasion, and Metastasis

The aforementioned anoikis studies illustrate the advantages of maintaining the iCa^2+^- dependent epithelial program rather than resorting to EMT. Experimental evidence has recently emerged that E-cadherin plays a positive and necessary role in metastasis of at least some breast cancers [151]. In diverse mouse models of invasive ductal carcinoma, Ewald’s group showed that deletion of E-cadherin enhanced initial invasiveness but reduced colony formation and metastasis [151]. Similar results were obtained by KD of E-cadherin in patient-derived xenografts. Mechanistically, they found that loss of E-cadherin upregulated TGF beta signaling and ROS formation leading to apoptosis. Interestingly, suppression of ROS with *N*-acetyl cysteine prevented apoptosis and restored metastatic tumor growth. Again, this finding will interest those who question the wisdom of the widespread use of antioxidant dietary supplements [152]. The generality of the conclusions is uncertain. Others have pointed out that disruption of the E-cadherin gene occurs in 10% of breast cancers, especially those of lobular origin, and at least some of them are capable of metastasis [153]. Thus, tissue origin may influence the route to metastasis as well as its efficiency. It should also be noted that the human tumors used in the Padmanaban study had been preselected for persistence of E-cadherin expression [151].

These and other studies have led to the idea that EMT is induced only at the invasive front of a tumor to liberate both single mesenchymal-like cells and clusters that either have not undergone EMT or have reversed a partial EMT [154,155,156]. However, some assumptions of this model too have been challenged. Using 3D cultured mouse mammary organoids, Shamir investigated the role of E-cadherin by inducibly deleting its gene in the presence or absence of ectopic Twist1, an EMT-associated transcription factor [157]. Surprisingly, they found that, instead of being repressed by Twist, E-cadherin was actually required for Twist1-induced single cell dissemination and could be found in complex with beta catenin at the plasma membrane of migrating, cytokeratin-positive cells.

It should be noted that E-cadherin can participate in antithetical programs, depending on its available partners, context, and localization [158,159]. Nevertheless, caution should be exercised in extrapolating from one experimental system to another. Cancer is infinitely heterogeneous. Cancer cells exploit whatever tools are available in their local environment, and outcomes may vary depending on whether the cells are squamous or glandular in origin, their dependence on growth factor receptor signaling as determined by protein biomarkers or RNA profiles, their mutational background, and their location in the primary tumor, circulation, or secondary site. Thus, a tumor of squamous origin with biomarkers/profile indicating dependence on EGFR signaling might be prevented from metastasizing by combining therapies that target EGFR signaling and iCa2+ signaling, while the combination might be counterproductive in another context. Experience with targeted therapy has taught us that this profiling is critical in determining whether a targeted therapy will extend patient survival or merely subject them to unnecessary toxicity and expense while depriving them of potentially more efficacious modalities.

## 7. Conclusions

Cytosolic Ca^2+^ is a multipotent tool whose regulation is crucial to all aspects of cell physiology. The frequent addiction of cancer cells to Ca^2+^ signaling has made it a prime target for chemotherapy [160,161]. However, inhibiting general Ca^2+^ storage and dispensation mechanisms can be broadly toxic to normal tissues such as cardiac muscle and undermine its tumor-suppressive functions as well. The last fifteen years have seen great strides in characterizing the molecular elements of Ca^2+^ signaling and their perturbations in discrete cancer subtypes. For Ca^2+^ manipulation to realize its potential as a precision tool in the anticancer arsenal, this process must be continued and refined to reveal cell type-specific targets whose inhibition does not impair major organ function. In addition, gene expression profiling and pathway analysis must become routine in the clinic to identify subtypes with a common Achilles’ heel.
